# Supplementary material for: A scoping review to map the concept, content, and outcome of wilderness programs for childhood cancer survivors
Source: PLoS One. 2021 Jan 6;16(1):e0243908. doi: 10.1371/journal.pone.0243908 (PMC7787391; doi:10.1371/journal.pone.0243908)
Supplement: S2 File — Results of the comprehensive search strategy in databases. (PDF) [file pone.0243908.s002.pdf]

S2 File. Results of the comprehensive search strategy in databases

Database	Records before deduplication	Records after deduplication
Amed	4	4
Cinahl	144	76
Cochrane Library	19	4
Embase	558	217
Eric	72	70
Google scholar	18	18
Medline (Ovid)	388	386
Psycinfo	52	25
Scopus	1101	748
Sociological Abstracts	0	0
Sportdiscus	3	0
Svemed+	0	0
Web of Science	597	298
Total	2956	**1846**

Medline (Ovid) - April 23, 2019

# ▲	Searches	Results
1	exp Neoplasms/	3159223
2	(neoplas* or cancer* or tumor or tumors or tumour* or carcinoma* or leukem* or leukaem* or malignan* or oncolog*).ti,ab.	3202751
3	1 or 2	4069431
4	exp Wilderness/ or exp Forests/ or exp Recreation Therapy/	7767
5	(((wilderness or adventure* or nature* or natural or open air* or outdoor* or recreational* or forest* or urban) adj3 (program* or therap* or treatment* or rehab* or training)) or (forest bath* or shinrin-yoku)).ti,ab.	14089
6	4 or 5	21697
7	exp Young Adult/ or exp CHILD/ or exp ADOLESCENT/ or exp Minors/ or PUBERTY/ or exp PEDIATRICS/	3229672
8	(Child* or Schoolchild* or School age* or Adoles* or Teen* or Boy* or Girl* or Minor* or Pubert* or Pubescen* or Prepubescen* or Pediatric* or Paediatric* or Peadiatric* or young adult* or aya).ti,ab.	1997186
9	7 or 8	3988876
10	3 and 6 and 9	387

Cinahl – April 24, 2019

#	Query	Results
S10	S3 AND S6 AND S9	144
S9	S7 OR S8	1,116,956
S8	TI (Child* OR Schoolchild* OR School age* OR Adoles* OR Teen* OR Boy* OR Girl* OR Minor* OR Pubert* OR Pubescen* OR Prepubescen* OR Pediatric* OR Paediatric* OR Peadiatric* OR young adult*) OR AB (Child* OR Schoolchild* OR School age* OR Adoles* OR Teen* OR Boy* OR Girl* OR Minor* OR Pubert* OR Pubescen* OR Prepubescen* OR Pediatric* OR Paediatric* OR Peadiatric* OR young adult*)	603,688
S7	(MH "Adolescence+" OR MH "Young Adult" OR MH "Child+" OR MH "Minors (Legal" OR MH "Puberty+" OR MH "Pediatrics+")	926,587
S6	S4 OR S5	10,290
S5	TI (((wilderness OR adventure* OR nature* OR natural OR open air* OR outdoor* OR recreational* OR forest* OR urban) N3 (program* OR therap* OR treatment* OR rehab* OR training)) OR (forest bath* OR shinrin-yoku)) OR AB (((wilderness OR adventure* OR nature* OR natural OR open air* OR outdoor* OR recreational* OR forest* OR urban) N3 (program* OR therap* OR treatment* OR rehab* OR training)) OR (forest bath* OR shinrin-yoku))	5,835
S4	(MH "Recreational Therapy" OR MH "Natural Environment" OR MH "Wilderness Experience")	4,828
S3	S1 OR S2	617,346
S2	TI (neoplas* OR cancer* OR tumor OR tumors OR tumour* OR carcinoma* OR leukem* OR leukaem* OR malignan* OR oncolog*) OR AB (neoplas* OR cancer* OR tumor OR tumors OR tumour* OR carcinoma* OR leukem* OR leukaem* OR malignan* OR oncolog*)	480,105
S1	(MH "Neoplasms+")	456,525

Scopus – April 24, 2019

#	Search	Results
4	#1 AND #2 AND #3	1,101
3	(TITLE-ABS-KEY ((wilderness OR adventure* OR nature* OR natural OR "open air*" OR outdoor* OR recreational* OR forest* OR urban) W/4 program*) OR TITLE-ABS-KEY ((wilderness OR adventure* OR nature* OR natural OR "open air*" OR outdoor* OR recreational* OR forest* OR urban) W/4 therap*) OR TITLE-ABS-KEY ((wilderness OR adventure* OR nature* OR natural OR "open air*" OR outdoor* OR recreational* OR forest* OR urban) W/4 treatment*) OR TITLE-ABS-KEY ((wilderness OR adventure* OR nature* OR natural OR "open air*" OR outdoor* OR recreational* OR forest* OR urban) W/4 rehab*) OR TITLE-ABS-KEY ((wilderness OR adventure* OR nature* OR natural OR "open air*" OR outdoor* OR	70,724

	recreational* OR forest* OR urban) W/4 training) OR TITLE-ABS-KEY ("forest bath*" OR shinrin-yoku))	
2	TITLE-ABS-KEY (child* OR schoolchild* OR "School age*" OR adoles* OR teen* OR boy* OR girl* OR minor* OR pubert* OR pubescen* OR prepubescen* OR pediatric* OR paediatric* OR peadiatric* OR "young adult*" OR aya)	5,303,706
1	(TITLE-ABS-KEY (neoplas* OR cancer* OR tumor OR tumors OR tumour* OR carcinoma* OR leukem* OR leukaem* OR malignan* OR oncolog*)	5,066,208

Eric – April 27, 2019

Eric does not permit truncation and due to the limited search possibilities, a simplified search was done in this database.

(wilderness OR adventure OR nature OR natural OR "open air" OR outdoor OR recreational OR forest OR urban OR "forest bath" OR shinrin-yoku) AND (neoplasm OR neoplasms OR cancer OR cancers OR tumor OR tumors OR tumour OR tumours OR carcinoma OR carcinomas OR leukemia OR leukemias OR leukemic OR leukaemia OR leukaemias OR leukaemic OR malignant OR malignancy OR malignancies OR oncologic OR oncology) AND (Child OR children OR Schoolchild OR schoolchildren OR "School age" OR "school aged" OR Adolescent OR adolescents OR adolescence OR Teen OR teens OR teenage OR teenager OR teenagers OR Boy OR boys OR Girl OR girls OR Minor OR minors OR Puberty OR Pubescent OR pubescents OR pubescence OR Prepubescent OR prepubescents OR prepubescence OR Pediatric OR pediatrics OR Paediatric OR paediatrics OR Peadiatric OR peadiatrics OR "young adult" OR "young adults" OR aya)

Embase – May 14, 2019

# ▼	Searches	Results
10	3 and 8 and 9	558
9	4 or 5	41385
8	6 or 7	5603903
7	(neoplas* or cancer* or tumor or tumors or tumour* or carcinoma* or leukem* or leukaem* or malignan* or oncolog*).ti,ab.	4503712
6	exp neoplasm/	4544167
5	(((wilderness or adventure* or nature* or natural or open air* or outdoor* or recreational* or forest* or urban) adj3 (program* or therap* or treatment* or rehab* or training)) or (forest bath* or shinrin-yoku)).ti,ab.	20421
4	wilderness/ or forest/ or exp recreational therapy/	21401

3	1 or 2	4108426
2	(young adult* or prime adult* or child* or adolescen* or minor or minors or juvenile* or puberty or pubescen* or pediatric* or paediatric* or schoolchild* or school age* or teen or teens or teenage* or boy* or girl* or prepuberty or prepubescen* or aya).ti,ab.	2743283
1	exp young adult/ or child/ or adolescent/ or exp "minor (person)"/ or puberty/ or pediatrics/	2932346

Psycinfo – May 14, 2019

# ▼	Searches	Results
10	7 and 8 and 9	52
9	3 or 4	79248
8	2 or 5	7979
7	1 or 6	921614
6	pediatrics/ or exp puberty/	25986
5	exp recreation therapy/ or exp wilderness experience/ or adventure therapy/ or therapeutic camps/	1471
4	exp Neoplasms/	48536
3	(neoplas* or cancer* or tumor or tumors or tumour* or carcinoma* or leukem* or leukaem* or malignan* or oncolog*).ti,ab.	76793
2	(((wilderness or adventure* or nature* or natural or open air* or outdoor* or recreational* or forest* or urban) adj3 (program* or therap* or treatment* or rehab* or training)) or (forest bath* or shinrin-yoku)).ti,ab.	6867
1	(Child* or Schoolchild* or School age* or Adoles* or Teen* or Boy* or Girl* or Minor* or Pubert* or Pubescen* or Prepubescen* or Pediatric* or Paediatric* or Peadiatric* or young adult* or aya).ti,ab.	920837

Web of Science – May 14, 2019

# 4	597	#3 AND #2 AND #1

# 3	5,998,009	TS=(Child* or Schoolchild* or School age* or Adoles* or Teen* or Boy* or Girl* or Minor* or Pubert* or Pubescen* or Prepubescen* or Pediatric* or Paediatric* or Peadiatric* or young adult* or aya)
# 2	6,289,655	TS=(neoplas* or cancer* or tumor or tumors or tumour* or carcinoma* or leukem* or leukaem* or malignan* or oncolog*)
# 1	30,038	TS=(wilderness NEAR/4 program*) OR TS=(wilderness NEAR/4 therap*) OR TS=(wilderness NEAR/4 therap*) OR TS=(wilderness NEAR/4 rehab*) OR TS=(wilderness NEAR/4 training*) OR TS=(adventure* NEAR/4 program*) OR TS=(adventure* NEAR/4 therap*) OR TS=(adventure* NEAR/4 therap*) OR TS=(adventure* NEAR/4 rehab*) OR TS=(adventure* NEAR/4 training*) OR TS=(nature* NEAR/4 program*) OR TS=(nature* NEAR/4 therap*) OR TS=(nature* NEAR/4 therap*) OR TS=(nature* NEAR/4 rehab*) OR TS=(nature* NEAR/4 training*) OR TS=(natural NEAR/4 program*) OR TS=(natural NEAR/4 therap*) OR TS=(natural NEAR/4 therap*) OR TS=(natural NEAR/4 rehab*) OR TS=(natural NEAR/4 training*) OR TS=("open air*" NEAR/4 program*) OR TS=("open air*" NEAR/4 therap*) OR TS=("open air*" NEAR/4 therap*) OR TS=("open air*" NEAR/4 rehab*) OR TS=("open air*" NEAR/4 training*) OR TS=(outdoor* NEAR/4 program*) OR TS=(outdoor* NEAR/4 therap*) OR TS=(outdoor* NEAR/4 therap*) OR TS=(outdoor* NEAR/4 rehab*) OR TS=(outdoor* NEAR/4 training*) OR TS=(recreational NEAR/4 program*) OR TS=(recreational NEAR/4 therap*) OR TS=(recreational NEAR/4 therap*) OR TS=(recreational NEAR/4 rehab*) OR TS=(recreational NEAR/4 training*) OR TS=(forest* NEAR/4 program*) OR TS=(forest* NEAR/4 therap*) OR TS=(forest* NEAR/4 therap*) OR TS=(forest* NEAR/4 rehab*) OR TS=(forest* NEAR/4 training*) OR TS=(urban NEAR/4 program*) OR TS=(urban NEAR/4 therap*) OR TS=(urban NEAR/4 therap*) OR TS=(urban NEAR/4 rehab*) OR TS=(urban NEAR/4 training*) OR TS=("forest bath*") OR TS=(shinrin-yoku)

Sportdiscus – May 14, 2019

#	Query	Resultaten
S4	S1 AND S2 AND S3	3
S3	(((((ZE "young adults")) or ((ZE "children"))) or ((ZE "pediatrics"))) or ((ZE "puberty"))) OR TI (Child* or Schoolchild* or "School age*" or Adoles* or Teen* or Boy* or Girl* or Minor* or Pubert* or Pubescen* or Prepubescen* or Pediatric* or Paediatric* or Peadiatric* or "young adult*" or aya) OR AB (Child* or Schoolchild* or "School age*" or Adoles* or Teen* or Boy* or Girl* or Minor* or Pubert* or Pubescen* or Prepubescen* or Pediatric* or Paediatric* or Peadiatric* or "young adult*" or aya)	137,116

S2	(((ZE "wilderness areas") or (ZE "wilderness areas -- recreational use") or (ZE "wilderness survival")) or ((ZE "recreational therapy") or (ZE "recreational therapy for children"))) OR TI ((wilderness or adventure* or nature* or natural or "open air*" or outdoor* or recreational* or forest* or urban) N/2 (program* or therap* or treatment* or rehab* or training)) OR AB ((wilderness or adventure* or nature* or natural or "open air*" or outdoor* or recreational* or forest* or urban) N/2 (program* or therap* or treatment* or rehab* or training)) OR TI ("forest bath*" or shinrin-yoku) OR AB ("forest bath*" or shinrin-yoku)	1,832
S1	((ZE "cancer") or (ZE "cancer in young adults")) OR TI (neoplas* or cancer* or tumor or tumors or tumour* or carcinoma* or leukem* or leukaem* or malignan* or oncolog*) OR AB (neoplas* or cancer* or tumor or tumors or tumour* or carcinoma* or leukem* or leukaem* or malignan* or oncolog*)	25,764

Cochrane Library – May 22, 2019

ID	Search	Hits
#1	(neoplas* or cancer* or tumor or tumors or tumour* or carcinoma* or leukem* or leukaem* or malignan* or oncolog*):ab,ti 1	187437
#2	((wilderness or adventure* or nature* or natural or "open air*" or outdoor* or recreational* or forest* or urban) NEAR/3 (program* or therap* or treatment* or rehab* or training)):ab,ti	1424
#3	("forest bath*" or shinrin-yoku):ab,ti	2
#4	("young adult*" or "prime adult*" or child* or adolescen* or minor or minors or juvenile* or puberty or pubescen* or pediatric* or paediatric* or schoolchild* or school age* or teen or teens or teenage* or boy* or girl* or prepuberty or prepubescen* or aya):ab,ti	156850
#5	#2 OR #3	1426
#6	#1 AND #4 AND #5	19

AMED May 13th, 2019 (saved search retrieved May 27th, 2019)

☐	S36	S7 AND S21 AND S33	**Search modes** - Find all my search terms	View Results (4)	View Details	Edit
☐	S35	S7 AND S21 AND S33	**Limiters** - Abstract Available<br>**Search modes** - Find all my search terms	View Results (4)	View Details	Edit
☐	S34	S7 AND S21 AND S33	**Search modes** - Find all my search terms	View Results (4)	View Details	Edit
☐	S33	S22 OR S23 OR S24 OR S25 OR S26 OR S32	**Search modes** - Find all my search terms	View Results (26,648)	View Details	Edit
☐	S32	S28 OR S29	**Search modes** - Find all my search terms	View Results (26,629)	View Details	Edit
☐	S31	(AB Child* OR Schoolchild* OR School age* OR Adoles* OR Teen* OR Boy* OR Girl* OR Minor* OR Pubert* OR Pubescen* OR Prepubescen* OR Pediatric* OR Paediatric* OR Peadiatric* OR young adult*)	**Search modes** - Find all my search terms	View Results (25,373)	View Details	Edit
☐	S30	(AB Child* OR Schoolchild* OR School age* OR Adoles* OR Teen* OR Boy* OR Girl* OR Minor* OR Pubert* OR Pubescen* OR Prepubescen* OR Pediatric* OR Paediatric* OR Peadiatric* OR young adult*) AND (S28 OR S29)	**Search modes** - Find all my search terms	View Results (21,525)	View Details	Edit
☐	S29	AB Child* OR Schoolchild* OR School age* OR Adoles* OR Teen* OR Boy* OR Girl* OR Minor* OR Pubert* OR Pubescen* OR Prepubescen* OR Pediatric* OR Paediatric* OR Peadiatric* OR young adult*	**Search modes** - Find all my search terms	View Results (18,035)	View Details	Edit
☐	S28	TI Child* OR Schoolchild* OR School age* OR Adoles* OR Teen* OR Boy* OR Girl* OR Minor* OR Pubert* OR Pubescen* OR Prepubescen* OR Pediatric* OR Paediatric* OR Peadiatric* OR young adult*	**Search modes** - Find all my search terms	View Results (17,722)	View Details	Edit
☐	S27	Child* OR Schoolchild* OR School age* OR Adoles* OR Teen* OR Boy* OR Girl*	**Search modes** - Find all my search terms	View Results (34,255)	View Details	Edit

		OR Minor* OR Pubert* OR Pubescen* OR Prepubescen* OR Pediatric* OR Paediatric* OR Peadiatric* OR young adult*		
☐	S26	(MM "Pediatrics+")	Search modes - Find all my search terms	View Results (15) | View Details | Edit
☐	S25	(MM "Puberty+")	Search modes - Find all my search terms	View Results (1) | View Details | Edit
☐	S24	(MM "Adolescence+")	Search modes - Find all my search terms	View Results (7) | View Details | Edit
☐	S23	(MM "Child+")	Search modes - Find all my search terms	View Results (225) | View Details | Edit
☐	S22	(MM "Young Adult")	Search modes - Find all my search terms	View Results (31) | View Details | Edit
☐	S21	S8 OR S9 OR S16 OR S20	Search modes - Find all my search terms	View Results (847) | View Details | Edit
☐	S20	S18 OR S19	Search modes - Find all my search terms	View Results (4) | View Details | Edit
☐	S19	AB forest bath* OR shinrin-yoku	Search modes - Find all my search terms	View Results (4) | View Details | Edit
☐	S18	TI forest bath* OR shinrin-yoku	Search modes - Find all my search terms	View Results (1) | View Details | Edit
☐	S17	forest bath* OR shinrin-yoku	Search modes - Find all my search terms	View Results (4) | View Details | Edit
☐	S16	S14 OR S15	Search modes - Find all my search terms	View Results (785) | View Details | Edit
☐	S15	AB S10 n3 S11	Search modes - Find all my search terms	View Results (644) | View Details | Edit
☐	S14	TI S10 n3 S11	Search modes - Find all my search terms	View Results (184) | View Details | Edit
☐	S13	S10 n3 S11	Search modes - Find all my search terms	View Results (785) | View Details | Edit
☐	S12	S10 AND S11	Search modes - Find all my search terms	View Results (8,154) | View Details | Edit
☐	S11	program* OR therap* OR treatment* OR rehab*	Search modes - Find all my search terms	View Results (206,359) | View Details | Edit
☐	S10	wilderness OR adventure* OR nature* OR natural OR open air* OR outdoor* OR recreational* OR forest* OR urban	Search modes - Find all my search terms	Rerun | View Details | Edit
☐	S9	"forests"	Search modes - Find all my search terms	Rerun | View Details | Edit
☐	S8	(MM "Wilderness Experience")	Search modes - Find all my search terms	Rerun | View Details | Edit
☐	S7	S4 OR S5 OR S6	Search modes - Find all my search terms	Rerun | View Details | Edit
☐	S6	AB neoplas* OR cancer* OR tumor OR tumors OR tumour* OR carcinoma* OR leukem* OR leukaem* OR malignan* OR oncolog*	Search modes - Find all my search terms	View Results (13,183) | View Details | Edit
☐	S5	TI neoplas* OR cancer* OR tumor OR tumors OR tumour* OR carcinoma* OR leukem* OR leukaem* OR malignan* OR oncolog*	Search modes - Find all my search terms	Rerun | View Details | Edit
☐	S4	"exp Neoplasms/" OR (MH "Neoplasms+")	Search modes - Find all my search terms	Rerun | View Details | Edit
☐	S3	"exp Neoplasms/"	Search modes - SmartText Searching	Rerun | View Details | Edit
☐	S2	"exp Neoplasms/"	Search modes - Find all my search terms	Rerun | View Details | Edit
☐	S1	exp Neoplasms/	Search modes - Boolean/Phrase	Rerun | View Details | Edit

Swemed+ May 13th, 2019

A simplified search was made since the search engine is very simple same as for ERIC

Based on the protocol:

(wilderness OR adventure OR nature OR natural OR "open air" OR outdoor OR recreational OR forest OR urban OR "forest bath" OR shinrin-yoku) AND (neoplasm OR neoplasms OR cancer OR cancers OR tumor OR tumors OR tumour OR tumours OR carcinoma OR carcinomas OR leukemia OR leukemias OR leukemic OR leukaemia OR leukaemias OR leukaemic OR malignant OR malignancy OR malignancies OR oncologic OR oncology) AND (Child OR children OR Schoolchild OR schoolchildren OR "School age" OR "school aged" OR Adolescent OR adolescents OR adolescence OR Teen OR teens OR teenage OR teenager OR teenagers OR Boy OR boys OR Girl OR girls OR Minor OR minors OR Puberty OR Pubescent OR pubescents OR pubescence OR Prepubescent OR prepubescents OR prepubescence OR Pediatric OR pediatrics OR Paediatric OR paediatrics OR Peadiatric OR peadiatrics OR "young adult" OR "young adults" OR aya)

Google scholar: 24 May, 2019

(cancer|tumor|tumour|carcinoma|leukemia|leukaemia|malignant|malignancy|malignancies|oncology)("young adults"|adolescents|child|puberty|aya|pediatric|paediatric)(wilderness|adventure|"nature programs"|"open air"|outdoor|forest|shinrin-yoku|"forest bath")

For practical reasons it was decided to select the publications that might be interesting for this scoping review. This is what was selected:

https://onlinelibrary.wiley.com/doi/epdf/10.1002/j.1556-6678.2002.tb00192.x

https://aee.memberclicks.net/assets/docs/SEER/SEER2017/2017%20final%20SEER%20booklet.pdf#page=19

http://climbingout.appstersdevelopment.com/wp-content/uploads/pdf/Slavin__Climbing_Out-%20Exploring_the_Psychosocial_Impacts_of_an_Adventure_Programme_for_Young_Adult_Survivors_of_Cancer.pdf

http://www2.cortland.edu/dotAsset/d447c0c5-0bb4-4f4c-a1e7-47a7c4247043.pdf#page=53

https://hub.hku.hk/handle/10722/249180

https://escholarship.org/uc/item/4r04g58z

https://www.hindawi.com/journals/ecam/2014/834360/abs/

https://scholarsbank.uoregon.edu/xmlui/handle/1794/12220

https://academic.oup.com/jpepsy/article-abstract/12/4/533/915419

https://digitalcommons.calpoly.edu/cgi/viewcontent.cgi?referer=https://scholar.google.nl/&httpsredir=1&article=1056&context=rptasp

https://academic.oup.com/jnci/article/92/20/1638/2905990

https://journals.sagepub.com/doi/abs/10.1177/105382590703000203

https://benthamopen.com/ABSTRACT/TOPSYJ-6-28

http://www.hdesignintegration.com/pdf/thesis.pdf

https://journals.sagepub.com/doi/abs/10.1177/1359105312465102

https://www.fs.fed.us/rm/pubs/rmrs_p066/rmrs_p066_127_133.pdf

https://bmccancer.biomedcentral.com/articles/10.1186/s12885-017-3366-6

http://citeseerx.ist.psu.edu/viewdoc/download?doi=10.1.1.458.552&rep=rep1&type=pdf
