# Supplementary material for: A scoping review to map the concept, content, and outcome of wilderness programs for childhood cancer survivors
Source: PLoS One. 2021 Jan 6;16(1):e0243908. doi: 10.1371/journal.pone.0243908 (PMC7787391; doi:10.1371/journal.pone.0243908)
Supplement: S3 File — (PDF) [file pone.0243908.s003.pdf]

S3 File. Standardized data extraction form
(Processed from Excel to Word format)

First author:	
Year of publication:	
Type of article	Quasi-experimental ☐<br>Qualitative ☐<br>Mixed-methods ☐<br>Text & Opinion ☐
Reviewer	MCJ ☐<br>WS ☐
Country:	
Funding source:	
Declaration of interest:	
Aim/objective	Primary aim:<br>Secondary aim:
Study design:	
Methodology:	
Participants	Inclusion criteria:<br>Exclusion criteria:<br>Number:<br>Age:<br>Gender:<br>Ethnicity:<br>Diagnosis:<br>Age at diagnosis:<br>Time after cancer treatment/diagnosis:<br>Stage of cancer (treatment/remission/relapse/palliative):<br>Previous cancer treatment:<br>Current medical treatment/other treatment:<br>Domestic situation (marital status, children, family situation):<br>Socio-economic status (education, income, work status):<br>Comorbidities:<br>Disabilities:

Program	Name:<br>Type (adventure/camping/wilderness):<br>Goals:<br>Length/duration:<br>Setting:<br>Camp- or expedition-based:<br>Open or a closed group structure:<br>Group size:<br>Number of facilitators per group:<br>Qualification of facilitators:<br>Costs:<br>Theoretical framework/concept:<br>Activities:<br>Psychotherapeutically work:<br>Role of nature:<br>Amount of structured/unstructured time:<br>Amount of time spent in group and individual activities:
Measurements/questionnaires:	
Health-related outcomes:	
Side-effects/risk factors:	
Ethical issues:	
Participation/support from family and friends:	
Incorporation program activities in daily life:	
Relationship WT programs and treatment institutions:	
Other important information/emergent themes:	
