# Supplementary material for: A scoping review to map the concept, content, and outcome of wilderness programs for childhood cancer survivors
Source: PLoS One. 2021 Jan 6;16(1):e0243908. doi: 10.1371/journal.pone.0243908 (PMC7787391; doi:10.1371/journal.pone.0243908)
Supplement: S4 File — Possible relevant articles in other languages. (PDF) [file pone.0243908.s004.pdf]

S4 File. Possible relevant articles in other languages

1. Crysdale J, Hershon L. [Surpassing oneself in order to cope... The therapeutic adventure]. Infirm Que. 2003;10(3):15-17.
2. Kondracka J. Assumptions of therapeutic recreation and its role according to the research carried out in the vacation recreation centre Barretstown for children suffering from chronic and life threatening illnesses. Psychiatr Psychol Klin. 2013;13(1):58-66
3. Tseitlin G, Valentei LV, Kozharskaia GV, Smirnova Zh V, Karpenko IA, Chekulaeva Iu V. [The main trends in a complex program of rehabilitation of children with oncological diseases]. Vestn Ross Akad Med Nauk. 2002(1):33-36
