# Supplementary material for: A scoping review to map the concept, content, and outcome of wilderness programs for childhood cancer survivors
Source: PLoS One. 2021 Jan 6;16(1):e0243908. doi: 10.1371/journal.pone.0243908 (PMC7787391; doi:10.1371/journal.pone.0243908)
Supplement: S5 File — Summary of included article characteristics. (PDF) [file pone.0243908.s005.pdf]

S5 File. Summary of included article characteristics

First Author	Country	Study design	Participants	Program	Measures	Key findings
Boren, 1985[1]	USA	Text & Opinion	Adolescent cancer survivors<br>Age: not reported<br>Number: n=30	Ski-rehabilitation program<br>Name: Adolescent Amputee Ski Rehabilitation Program<br>Duration: 1 week	Narrative description	The program enables participants to do a sport again by facilitating adaptation to the handicap.
Carlson, 2007[2]	USA	Text & Opinion	Children with serious, life-threating illnesses, including cancer<br>Age: 7-15 years<br>Number: not reported	Adventure therapy<br>Name: The Hole in the Wall Gang Camp<br>Duration: not reported	Narrative review	The adventure program is efficacious in helping participants regain and increase in sense of self-efficacy and personal agency, development of a sense of social responsibility, and confidence to persevere in situations that produce anxiety.
Dasson, 1982[3]	USA	Text & Opinion	Childhood cancer survivors<br>Age: 8-18 years<br>Number: n=294	Cancer camp<br>Name: Camp Good Days and Special Times<br>Duration: 5-10 days	Narrative description	This program provides a normal camping experience for participants and supports social interaction, learning new skills, and to gain self-confidence.
Epstein, 2004[4]	Canada	Text & Opinion	Adolescent cancer survivors<br>Age: not reported<br>Number: not reported	Adventure therapy<br>Name: not reported<br>Duration: not reported	Narrative review	Adventure therapy enhances adolescents' self-concepts: self-evaluation, self-exploration, self-reevaluation, self-acceptance, self-realization.
Gill, 2016[5]	USA	Quasi-experimental (wait-list control)	Young adult cancer survivors<br>Age: 18–39 years<br>Number: adventure therapy; n=50, control group; n=66	Adventure therapy<br>Name: First Descents<br>Duration: 1 week	Physical activity recall, sedentary behavior, Sallis self-efficacy, Exercise habits survey, Environmental-Change Self-Efficacy questionnaire, the Preferred Activities Questionnaire, The Enjoyment of Physical Activity Questionnaire, The Enjoyment of Inactive Recreation scale.	Adventure therapy significantly increases the level of physical activity during camp and three months after termination, although effects were attenuated over time.

Kessel, 1985[6]	USA	Mixed-methods	Chronically ill, physically disabled and able-bodied adolescents, among them adolescents with cancer<br>Age: not reported<br>Number: n=3 or 4 with cancer	Wilderness experience<br>Name: Adventure etc.<br>Duration: 9 days wilderness; 5 days in the city	Offer Self-Image Questionnaire for Adolescents, Nowicki-Strickland Personal Reaction Survey, Moos Family Environment Scale and interviews, 6 months post-program.	After the program, the chronically ill/disabled participants show a significant increase in internal locus of control. No changes were found in the able-bodied group. Both groups showed a significant improvement in body image after the program.
Paquette, 2017[7]	Canada	Mixed-methods	Adolescent cancer survivors<br>Age: 14-20 years<br>Number: n=52	Adventure therapy<br>Name: On the tip of the toes<br>Duration: 10 days	Rosenberg self-esteem questionnaire, Psychiatric Symptoms Index, Inventory of Parent and Peer attachment, Social desirability, and interviews.	The program significantly improved self-esteem and perceived quality of the relationship with parents and peers, with small to moderate effects. Improvement in self-esteem was maintained one year after follow-up. The qualitative results indicate that the program is favorable to the development of self-determination.
Pearson, 1989[8]	USA	Text & Opinion	Adolescent cancer survivors<br>Age: not reported<br>Number: not reported	Wilderness experience<br>Name: Breckenridge Outdoor Education Center (BOEC)<br>Duration: not reported	Narrative description	The experience has demonstrated to be a successful program to challenge participants and provide tools and skills to move on in their lives.
Rosenberg, 2014[9]	USA	Quasi-experimental (wait-list control)	Young adult cancer survivors<br>Age: 18–39 years<br>Number: adventure therapy first program; n=87, adventure therapy second program; n=41, control group; n=71	Adventure therapy<br>Name: First Descents<br>Duration: 6 days	Body Image Scale, Self-Compassion Scale-Short Form, Psychological Screening Inventory.	Compared to a wait-list control group, participants who took part in the program for the first time had improved body image, self-compassion and self-esteem, and less depression and alienation. Participants who took part for the second time, though also helped by the program in similar ways, were not better of psychologically than participants who took part for the first time.
Slavin, 2015[10]	UK	Qualitative	Those receiving a diagnosis of cancer during childhood or adolescence<br>Age: 18-30 years<br>Number: n=7	Adventure therapy<br>Name: Climbing out<br>Duration: 5 days	Semi-structured interviews, 1-3 years after the adventure program.	Several years after completion of the program, a return to normality in self-concept, a renewed sense of hope, cultivating self-efficacy, awareness & acceptance, and a supportive group is reported.

Stevens, 2004[11]	Canada	Qualitative	Adolescent cancer survivors<br>Age: 15-18 years<br>Number: n=11	Adventure therapy<br>Name: On the tip of the toes<br>Duration: 10 days	Unstructured interviews.	Four themes were reported upon participation in adventure therapy: developing connections, togetherness, rebuilding self-esteem, and creating memories.
Wagner, 2014[12]	USA	Qualitative	Young adult cancer survivors<br>Age: 18-39 years<br>Number: n=70	Adventure therapy<br>Name: First Descents<br>Duration: 1 week	Interviews	Awareness, connection, challenge, new perspectives, and support were the most common consequences reported upon participating in the program. Of all values, warm relationships with others, transference, and self-awareness had the most responses among participants.
Wingler[13]	USA	Qualitative	Certified recreational therapists<br>Age: not reported<br>Number: n=2	Adventure therapy<br>Name: CoachArt<br>Duration: 1 day in the winter, 2 days in the summer	Semi-structured interviews	The four major themes generated from this study were gaining control while losing control, pushing the limits here equals pushing the limits there, special but not different, and getting your life back only better.
Wynn, 2012[14]	New Zealand	Mixed-methods	Adolescent cancer survivors<br>Age: 17-21 years<br>Number: 5	Adventure therapy<br>Name: not reported<br>Duration: 8 days	Wagnild and Young's Resilience Scale, Group Cohesion measure. Semi-structured interviews, 4 months after the journey.	Trend for increased resilience after compared to before the journey (increased independence, personal pride, self-esteem, self-determination). The qualitative outcomes after the program were being one-self, group support and friendship, a little bit of pride, and back to reality.
Zebrack, 2017[15]	USA	Quasi-experimental	Young adult cancer survivors<br>Age: 18-40 years<br>Number: Pre-trip; n=247, Post-trip; n=196 , one month after trip; n=159.	Adventure therapy<br>Name: First Descents<br>Duration: 1 week	Patient Health Questionnaire-4, Cancer Behavior Inventory, Duke-UNC Functional Social Support Questionnaire.	All participants demonstrated significant improvement in self-efficacy over time. Distressed participants reported a significantly greater decrease in distress symptoms and greater increase in self-efficacy and social support at post-trip and 1 months later when compared to non-distressed participants.

1. Boren HA, Meell H. Adolescent amputee ski rehabilitation program. *Journal of the Association of Pediatric Oncology Nurses.* 1985;2(1):16-23.

2. Carlson KP, Cook M. Challenge by Choice: Adventure-Based Counseling for Seriously Ill Adolescents. *Child Adolesc Psychiatr Clin North Am.* 2007;16(4):909-919.
3. Dasson ME. A chance to be normal again... camp for children with cancer -- Camp Good Days and Special Times. *Cancer Nursing.* 1982;5(6):453-459.
4. Epstein I. Adventure therapy: a mental health promotion strategy in pediatric oncology. *J Pediatr Oncol Nurs.* 2004;21(2):103-110.
5. Gill E, Goldenberg M, Starnes H, Phelan S. Outdoor adventure therapy to increase physical activity in young adult cancer survivors. *J Psychosoc Oncol.* 2016;34(3):184-199.
6. Kessell M, Resnick MD, Blum RW. Adventure, Etc.—A health-promotion program for chronically ill and disabled youth. *Journal of Adolescent Health Care.* 1985;6(6):433-438.
7. Paquette L, Fortin J, Crete A, Maltais D, Brassard A. Effect of an outdoord developmental adventure program on the psychosocial adjustments of adolescents journeying with cancer. Paper presented at: *Proceedings of the 2017 Symposium on Experiential Education Research* - 45nd Annual International AEE Conference2017; Montreal, Canada.
8. Pearson J. A wilderness program for adolescents with cancer. *Journal of the Association of Pediatric Oncology Nurses.* 1989;6(2):24-25.
9. Rosenberg RS, Lange W, Zebrack B, Moulton S, Kosslyn SM. An outdoor adventure program for young adults with cancer: positive effects on body image and psychosocial functioning. *J Psychosoc Oncol.* 2014;32(5):622-636.
10. Slavin M. *Climbing Out: Exploring the Psychosocial Impacts of an Adventure Programme for Young Adult Survivors of Cancer*, University of Glasgow; 2015.
11. Stevens B, Kagan S, Yamada J, et al. Adventure therapy for adolescents with cancer. *Pediatric Blood & Cancer.* 2004;43(3):278-284.
12. Wagner A. An examination of the benefits that adventure and wilderness therapy has on young adult cancer fighthers and survivors. https://digitalcommons.calpoly.edu/cgi/viewcontent.cgi?referer=https://scholar.google.nl/&httpsredir=1&article=1056&context=rptasp: the Faculty of the Recreation, Parks, & Tourism Administration Department, California Polytechnic State University, San Luis Obispo; 2014.
13. Wingler D. *Bringing Adventure-Based Therapy to Adolescent Cancer Patients: Design principles for interior oncology environments*.
14. Wynn B, Frost A, Pawson P. Adventure therapy proves successful for adolescent survivors of childhood cancers. *Nurs N Z.* 2012;18(1):28-30.
15. Zebrack B, Kwak M, Sundstrom L. First Descents, an adventure program for young adults with cancer: who benefits? *Supportive Care in Cancer.* 2017;25(12):3665-3673.
