# Supplementary material for: A scoping review to map the concept, content, and outcome of wilderness programs for childhood cancer survivors
Source: PLoS One. 2021 Jan 6;16(1):e0243908. doi: 10.1371/journal.pone.0243908 (PMC7787391; doi:10.1371/journal.pone.0243908)
Supplement: S6 File — Health-related outcomes of wilderness therapy. * (n) = the number of articles in which the outcome is reported. (PDF) [file pone.0243908.s006.pdf]

S6 File. Health-related outcomes of wilderness therapy

Health domains	Outcomes (n)*	References
Physical health	Increase in:	
	Physical activity (4)	5-7,10
	Illness management (1)	13
	Self-care (1)	13
	Decrease in:	
	Discomfort (2)	7,9
	TV time (1)	5
	Sitting time (1)	5
	Fatigue/low energy (1)	9
Mental health	Increase in:	
	Self-esteem (5)	7,9,11,12,14
	Self-efficacy (4)	2,6,10,15
	Self-confidence (4)	2,3,7,13
	Achievements (3)	1,7,12
	Independence (3)	7,13,14
	Self-concept (3)	4,10,14
	Feeling normal (3)	1,3,13
	Overcoming challenges (3)	1,8,12
	New perspectives (3)	8,12,13
	Body image (2)	9,13
	Hope (2)	10,12
	Self-awareness (2)	10,12
	Enjoyment (2)	11,12
	Empowerment (2)	11,12
	Control (2)	13,14
	Letting go (2)	12,13
	Self-compassion (1)	9
	Assertiveness (1)	13
	Expressing emotions (1)	7
	Personal pride (1)	14
	Amazement with nature (1)	7
	Acceptance (1)	10
	Feeling healthy (1)	12
	Decrease in:	
	Psychological distress (2)	7,15
	Alienation (2)	7,9
	Depression (1)	9
	Depressive feelings (1)	9
	Somatic anxiety (1)	9
	Memory/concentration problems (1)	9
Social health	Increase in:	
	Social involvement (5)	3,7,11-13
	Social support (4)	10,12,14,15
	Feeling of togetherness (1)	11
	Social responsibility (1)	2

* (n) = the number of articles in which the outcome is reported

1. Boren HA, Meell H. Adolescent amputee ski rehabilitation program. *Journal of the Association of Pediatric Oncology Nurses.* 1985;2(1):16-23.
2. Carlson KP, Cook M. Challenge by Choice: Adventure-Based Counseling for Seriously Ill Adolescents. *Child Adolesc Psychiatr Clin North Am.* 2007;16(4):909-919.
3. Dasson ME. A chance to be normal again... camp for children with cancer -- Camp Good Days and Special Times. *Cancer Nursing.* 1982;5(6):453-459.
4. Epstein I. Adventure therapy: a mental health promotion strategy in pediatric oncology. *J Pediatr Oncol Nurs.* 2004;21(2):103-110.
5. Gill E, Goldenberg M, Starnes H, Phelan S. Outdoor adventure therapy to increase physical activity in young adult cancer survivors. *J Psychosoc Oncol.* 2016;34(3):184-199.
6. Kessell M, Resnick MD, Blum RW. Adventure, Etc.—A health-promotion program for chronically ill and disabled youth. *Journal of Adolescent Health Care.* 1985;6(6):433-438.
7. Paquette L, Fortin J, Crete A, Maltais D, Brassard A. Effect of an outdoord developmental adventure program on the psychosocial adjustments of adolescents journeying with cancer. Paper presented at: *Proceedings of the 2017 Symposium on Experiential Education Research* - 45nd Annual International AEE Conference2017; Montreal, Canada.
8. Pearson J. A wilderness program for adolescents with cancer. *Journal of the Association of Pediatric Oncology Nurses.* 1989;6(2):24-25.
9. Rosenberg RS, Lange W, Zebrack B, Moulton S, Kosslyn SM. An outdoor adventure program for young adults with cancer: positive effects on body image and psychosocial functioning. *J Psychosoc Oncol.* 2014;32(5):622-636.
10. Slavin M. *Climbing Out: Exploring the Psychosocial Impacts of an Adventure Programme for Young Adult Survivors of Cancer*, University of Glasgow; 2015.
11. Stevens B, Kagan S, Yamada J, et al. Adventure therapy for adolescents with cancer. *Pediatric Blood & Cancer.* 2004;43(3):278-284.
12. Wagner A. An examination of the benefits that adventure and wilderness therapy has on young adult cancer fighthers and survivors. https://digitalcommons.calpoly.edu/cgi/viewcontent.cgi?referer=https://scholar.google.nl/&httpsredir=1&article=1056&context=rptasp: the Faculty of the Recreation, Parks, & Tourism Administration Department, California Polytechnic State University, San Luis Obispo; 2014.
13. Wingler D. *Bringing Adventure-Based Therapy to Adolescent Cancer Patients: Design principles for interior oncology environments*.
14. Wynn B, Frost A, Pawson P. Adventure therapy proves successful for adolescent survivors of childhood cancers. *Nurs N Z.* 2012;18(1):28-30.
15. Zebrack B, Kwak M, Sundstrom L. First Descents, an adventure program for young adults with cancer: who benefits? *Supportive Care in Cancer.* 2017;25(12):3665-3673.
