# Supplementary material for: A scoping review to map the concept, content, and outcome of wilderness programs for childhood cancer survivors
Source: PLoS One. 2021 Jan 6;16(1):e0243908. doi: 10.1371/journal.pone.0243908 (PMC7787391; doi:10.1371/journal.pone.0243908)
Supplement: S7 File — Assessment of the methodological quality of each included article. Assessed according to the following checklists: 1The Joanna Briggs Institute (2019): Critical Appraisal tools for use in JBI Systematic Reviews. 2MMAT (2019): Mixed Methods Appraisal Tool. (PDF) [file pone.0243908.s007.pdf]

S7 File. Assessment of the methodological quality of each included article

**Text & Opinion**[1]	1. Is the source of the opinion clearly identified?	2. Does the source of opinion have standing in the field of expertise	3. Are the interests of the relevant population the central focus of the opinion?	4. Is the stated position the result of an analytical process, and is there logic in the opinion expressed?	5. Is there reference to the extant literature?	6. Is any incongruence with the literature/sources logically defended				
Boren, 1985	Yes	Yes	Yes	Yes	No	No				
Carlson, 2007	Yes	Yes	Yes	Yes	Yes	Yes				
Dasson, 1982	Yes	Yes	Yes	Yes	No	No				
Epstein, 2004	Yes	Yes	Yes	Yes	Yes	Yes				
Pearson, 1989	Yes	Yes	Yes	Yes	No	No				

**Qualitative**[1]	1. Congruity between the stated philosophical perspective and the research methodology?	2. Congruity between the research methodology and the research question or objectives?	3. Congruity between the research methodology and the methods used to collect data?	4. Congruity between the research methodology and the representation and analysis of data?	5. Congruity between the research methodology and the interpretation of results?	6. Is there a statement locating the researcher culturally or theoretically?	7. Is the influence of the researcher on the research, and vice-versa, addressed?	8. Are participants, and their voices, adequately represented?	9. Research ethical according to current criteria or, for recent studies, and evidence of ethical approval by an appropriate body?	10. Do the conclusions drawn in the research report flow from the analysis, or interpretation, of the data?
Slavin, 2015	Yes	Yes	Yes	Yes	Yes	Yes	Yes	Yes	Yes	Yes
Stevens, 2004	Yes	Yes	Yes	Yes	Yes	No	No	Yes	Yes	Yes
Wagner, 2014	Yes	Yes	Yes	Yes	Yes	No	No	No	No	Yes
Wingler	Yes	Yes	Yes	Yes	Yes	No	No	Yes	Yes	Yes

**Mixed-methods[2]**	1. Are there clear research questions?	2. Do the collected data allow to address the research questions?	3. Is there an adequate rationale for using a mixed-methods design to address the research questions?	4. Are the different components of the study effectively integrated to answer the research questions?	5. Are the outputs of the integration of qualitative and quantitative components adequately interpreted?	6. Are divergences and inconsistencies between quantitative and qualitative results adequately addressed?	7. Do the different components of the study adhere to the quality criteria of each of the methods involved?			
Kessel, 1985	No	No	No	Yes	No	No	?			
Paquette, 2017	Yes	Yes	No	No	No	No	Yes			
Wynn, 2012	No	No	No	No	Yes	No	?			

**Quasi-experimental[1]**	1. Is it clear in the study what is the 'cause' and what is the 'effect'?	2. Were the participants included in any comparisons similar?	3. Were the participants included in any comparisons receiving similar treatment/care, other than the exposure or intervention of interest?	4. Was there a control group?	5. Were there multiple measurements of the outcome both pre and post the intervention/exposure?	6. Was follow up complete and if not, were differences between groups in terms of their follow up adequately described and analyzed?	7. Were the outcomes of participants included in any comparisons measured in the same way?	8. Were outcomes measured in a reliable way?	9. Was appropriate statistical analysis used?	
Gill, 2016	Yes	Yes	Yes	Yes	Yes	Yes	Yes	Yes	Yes	
Rosenberg, 2014	Yes	Yes	Yes	Yes	Yes	Yes	Yes	Yes	Yes	
Zebrack, 2017	Yes	Yes	Yes	No	Yes	Yes	Yes	Yes	Yes	

Assessed according to the following checklists: [1]The Joanna Briggs Institute (2019): Critical Appraisal tools for use in JBI Systematic Reviews. [2]MMAT (2019): Mixed Methods Appraisal Tool.

? = refers to Unclear/Can't tell

References:

Boren, H. A., & Meell, H. (1985). Adolescent amputee ski rehabilitation program. *Journal of Pediatric Oncology Nursing, 2*(1), 16-23.

Carlson, K. P., & Cook, M. (2007). Challenge by Choice: Adventure-Based Counseling for Seriously Ill Adolescents. *Child and Adolescent Psychiatric Clinics of North America, 16*(4), 909-919. doi:10.1016/j.chc.2007.05.002

Dasson, M. E. (1982). A chance to be normal again... camp for children with cancer -- Camp Good Days and Special Times. *Cancer Nursing, 5*(6), 453-459.

Epstein, I. (2004). Adventure therapy: a mental health promotion strategy in pediatric oncology. *Journal of Pediatric Oncology Nursing, 21*(2), 103-110.

Gill, E., Goldenberg, M., Starnes, H., & Phelan, S. (2016). Outdoor adventure therapy to increase physical activity in young adult cancer survivors. *Journal of Psychosocial Oncology, 34*(3), 184-199. doi:10.1080/07347332.2016.1157718

Kessell, M., Resnick, M. D., & Blum, R. W. (1985). Adventure, Etc.—A health-promotion program for chronically ill and disabled youth. *Journal of Adolescent Health Care, 6*(6), 433-438. doi:10.1016/s0197-0070(85)80048-6

Paquette, L., Fortin, J., Crete, A., Maltais, D., & Brassard, A. (2017). *Effect of and outdoor developmental adventure program on the psychosocial adjustment of adolescents journeying with cancer.* Paper presented at the Proceedings of the 2017 Symposium on Experiential Education Research - 45nd Annual International AEE Conference, Montreal, Canada.

Pearson, J. (1989). A wilderness program for adolescents with cancer. *Journal of the Association of Pediatric Oncology Nurses, 6*(2), 24-25.

Rosenberg, R. S., Lange, W., Zebrack, B., Moulton, S., & Kosslyn, S. M. (2014). An outdoor adventure program for young adults with cancer: positive effects on body image and psychosocial functioning. *Journal of Psychosocial Oncology, 32*(5), 622-636. doi:10.1080/07347332.2014.936652

Slavin, M. (2015). *Climbing Out: Exploring the Psychosocial Impacts of an Adventure Programme for Young Adult Survivors of Cancer.* (MSc in Psychological Studies), University of Glasgow. Retrieved from http://climbingout.appstersdevelopment.com/wp-content/uploads/pdf/Slavin__Climbing_Out-%20Exploring_the_Psychosocial_Impacts_of_an_Adventure_Programme_for_Young_Adult_Survivors_of_Cancer.pdf (2161992S)

Stevens, B., Kagan, S., Yamada, J., Epstein, I., Beamer, M., Bilodeau, M., & Baruchel, S. (2004). Adventure therapy for adolescents with cancer. *Pediatric Blood & Cancer, 43*(3), 278-284. doi:10.1002/pbc.20060

Wagner, A. (2014). *An examination of the benefits that adventure therapy and wilderness therapy has on young adult cancer fighters and survivors.* (Bachelor), California Polytechnic State University, San Luis Obispo, https://digitalcommons.calpoly.edu/cgi/viewcontent.cgi?referer=https://scholar.google.nl/&httpsredir=1&article=1056&context=rptasp

Wingler, D. *Bringing Adventure-Based Therapy to Adolescent Cancer Patients: Design principles for interior oncology environments.* (Master of Science and Design). Retrieved from http://www.hdesignintegration.com/pdf/thesis.pdf

Wynn, B., Frost, A., & Pawson, P. (2012). Adventure therapy proves successful for adolescent survivors of childhood cancers. *Nursing New Zealand (Wellington), 18*(1), 28-30.

Zebrack, B., Kwak, M., & Sundstrom, L. (2017). First Descents, an adventure program for young adults with cancer: who benefits? *Supportive Care in Cancer, 25*(12), 3665-3673. doi:10.1007/s00520-017-3792-7
